# A preclinical platform for assessing long-term drug efficacy exploiting mechanically tunable scaffolds colonized by a three-dimensional tumor microenvironment

**DOI:** 10.1186/s40824-023-00441-3

**Published:** 2023-10-18

**Authors:** Elly De Vlieghere, Koen Van de Vijver, Eva Blondeel, Nathan Carpentier, Rouba Ghobeira, Jarne Pauwels, Sebastian Riemann, Manon Minsart, Charlotte Fieuws, Johanna Mestach, Ans Baeyens, Nathalie De Geyter, Charlotte Debbaut, Hannelore Denys, Benedicte Descamps, Kathleen Claes, Anne Vral, Jo Van Dorpe, Kris Gevaert, Bruno G. De Geest, Wim Ceelen, Sandra Van Vlierberghe, Olivier De Wever

**Affiliations:** 1 Department of Human Structure and Repair, Laboratory of Experimental Cancer Research, Ghent University, Ghent, Belgium; 2 Polymer Chemistry and Biomaterials Group, Centre of Macromolecular Chemistry, Ghent University, Ghent, Belgium; 3 Cancer Research Institute Ghent (CRIG), Ghent University, Ghent, Belgium; 4 Department of Diagnostic Sciences, Ghent University Hospital, Ghent, Belgium; 5 Department of Applied Physics, Research Unit Plasma Technology (RUPT), Ghent University, Ghent, Belgium; 6 Department of Biomolecular Medicine, VIB Center for Medical Biotechnology, Ghent University, Ghent, Belgium; 7 Department of Biomolecular Medicine, Center for Medical Genetics, Ghent University, Ghent, Belgium; 8 Department of Human Structure and Repair, Radiobiology Group, Ghent University, Ghent, Belgium; 9 Department of Electronics and Information Systems, IBiTech-Biommeda, Ghent University, Ghent, Belgium; 10 Department of Medical Oncology, Ghent University Hospital, Ghent, Belgium; 11 Department of Electronics and Information Systems, IbiTech-Medisip, Ghent University, Ghent, Belgium; 12 Department of Pharmaceutics, Ghent University, Ghent, Belgium; 13 Department of Human Structure and Repair, Experimental Surgery Lab, Ghent University, Ghent, Belgium

**Keywords:** 3D cancer model, Long-term, Drug evaluation, Pre-clinical, Micro-environment, Stiffness

## Abstract

**Background:**

Long-term drug evaluation heavily relies upon rodent models. Drug discovery methods to reduce animal models in oncology may include three-dimensional (3D) cellular systems that take into account tumor microenvironment (TME) cell types and biomechanical properties.

**Methods:**

In this study we reconstructed a 3D tumor using an elastic polymer (acrylate-endcapped urethane-based poly(ethylene glycol) (AUPPEG)) with clinical relevant stiffness. Single cell suspensions from low-grade serous ovarian cancer (LGSOC) patient-derived early passage cultures of cancer cells and cancer-associated fibroblasts (CAF) embedded in a collagen gel were introduced to the AUPPEG scaffold. After self-organization in to a 3D tumor, this model was evaluated by a long-term (> 40 days) exposure to a drug combination of MEK and HSP90 inhibitors. The drug-response results from this long-term in vitro model are compared with drug responses in an orthotopic LGSOC xenograft mouse model.

**Results:**

The in vitro 3D scaffold LGSOC model mimics the growth ratio and spatial organization of the LGSOC. The AUPPEG scaffold approach allows to test new targeted treatments and monitor long-term drug responses. The results correlate with those of the orthotopic LGSOC xenograft mouse model.

**Conclusions:**

The mechanically-tunable scaffolds colonized by a three-dimensional LGSOC allow long-term drug evaluation and can be considered as a valid alternative to reduce, replace and refine animal models in drug discovery.

**Graphical Abstract:**

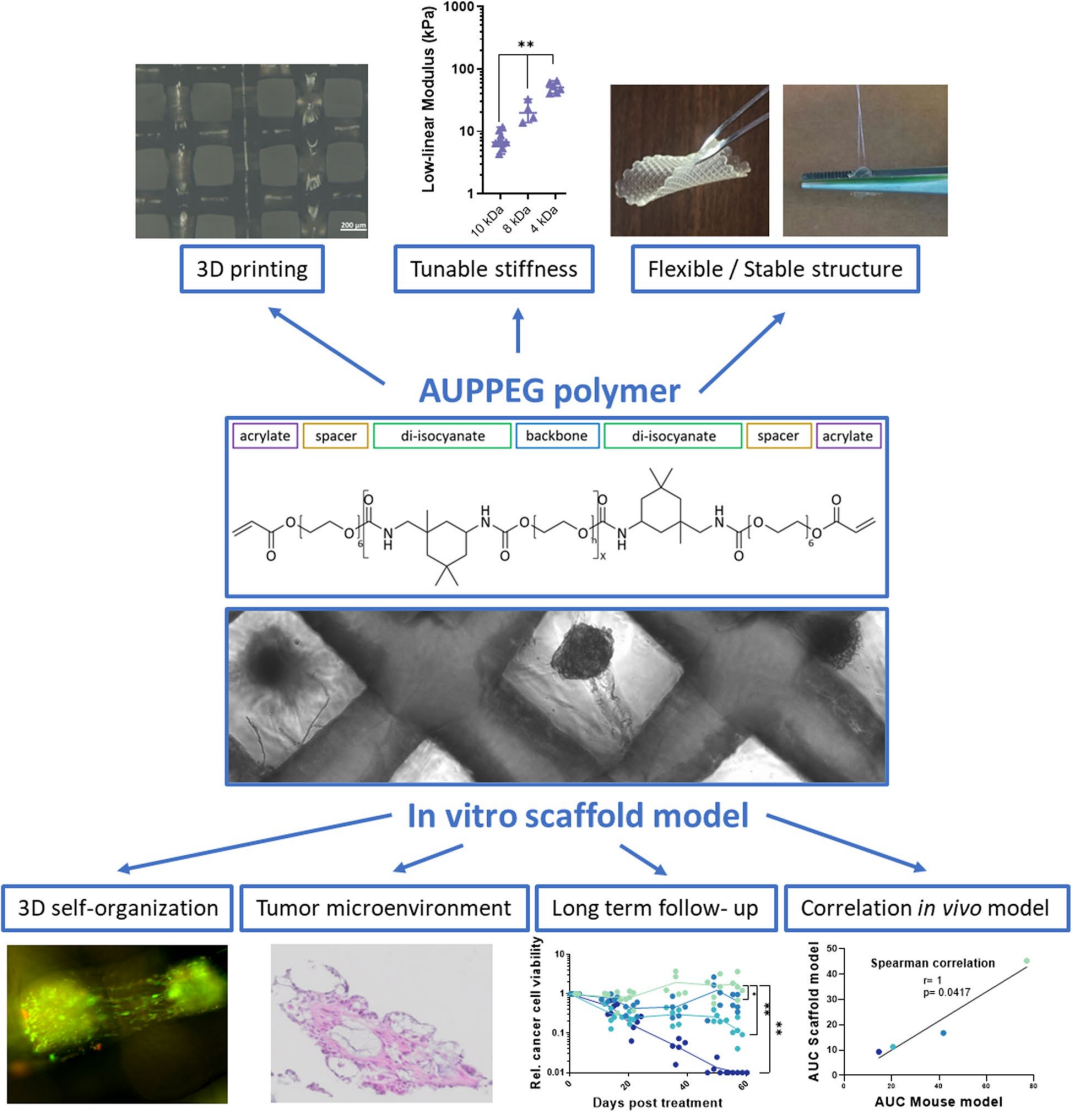

**Supplementary Information:**

The online version contains supplementary material available at 10.1186/s40824-023-00441-3.

## Introduction

Rodent models are heavily relied upon in oncology preclinical development. In Europe alone over 500,000 rodents are yearly used for basic oncology research [1]. In addition, long-term drug efficacy can often only be demonstrated in animal models, and that is also why authorities responsible for licensing drugs still insist on them as part of the approval process. Advances in in vitro technologies may bring the prospect of a reduction in the number of animals used, as well as an opportunity to develop better predictive tools to address the issues of drug attrition and resistance. However, the challenge will be to design models that hold significant advantages over current approaches. That means generating models that give robust and reproducible data that are predictive of human biology, and that allow long-term evaluation, needed to evaluate potential drug resistance issues. Ideally, all these properties should be combined taking into account a minimum of incremental costs.

Three-dimensional (3D) cellular models, including spheroids, show gradients of nutrients, and oxygen and drug supply leading to spatial heterogeneity in cell responses including induction of resistance as such reflecting the heterogeneity found in in vivo tumors. Although still simplified models, spheroids are increasingly used as biomimetic in vitro models of tumor tissues. Their scalability promoted academic and industrial interest particularly to evaluate drug responses. In addition, there is an increased awareness that elements from the tumor microenvironment (TME), such as cancer-associated fibroblast (CAF), contribute to therapy response [2–4]. Heterocellular spheroids combine cancer cells with CAF and show more and more use in preclinical research [5–7]. Most spheroid assays evaluate drug efficacy using a time span of one to two weeks [8–10], making it difficult to identify drug resistance. Long-term drug response evaluation is in general being performed using rodent models in which tumor engineering [11] and orthotopic patient-derived xenografts [12] ensure clinical relevance. 3D in vitro models that allow long-term evaluation of cancer therapy are being developed but are still limited (Table S1). Some of these models include TME cell types, others however do not take into account the biomechanical properties of a tumor. Cancer tissue is often recognized as stiffer than normal or adjacent tissues in various types of organs and these biomechanical cues play a role in tumor physiology and consequently drug responses. Intriguingly, only a limited number of studies use 3D cancer models taking into account TME, tissue-relevant stiffness and drug exposure times of 4 weeks or longer [13–15]. This long term-exposure is necessary to evaluate durability of treatment effectiveness and initiation of drug resistance.

Low-grade serous ovarian cancer (LGSOC) is a rare subtype of epithelial ovarian cancer and is characterized by a younger age at diagnosis. Patients with LGSOC are usually diagnosed in advanced stages and only 10–20% of such patients have more than 10 years survival after diagnosis. LGSOC is characterized by a high frequency of oncogenic mutations in KRAS, NRAS and BRAF in which approximately two thirds of the tumors have a mutually exclusive mutation in one of these genes [16–18]. Cytoreductive surgery, followed by platinum/paclitaxel chemotherapy is the most often used treatment option for patients with LGSOC. However, LGSOC is relatively resistant to standard chemotherapy, likely due to its low proliferative activity in comparison with other ovarian tumor types such as high-grade serous ovarian cancer (HGSOC). The oncogenic mutations affecting the MAPK pathway led to the evaluation of targeted agents, such as MEK inhibitors (MEKi). The recent phase II/III trial GOG281/LOGS showed a significant improved progression-free survival by the MEKi Trametinib compared to standard-of-care treatment (respectively 13.0 months vs 7.2 months) [19]. While very promising, these results highlight the occurrence of MEKi resistance and a clear need for more durable therapies. Unfortunately, the limited availability of preclinical models that allow long-term follow-up is a major restrain on innovative translational research, including in LGSOC (Table S1).

**Fig. 1:**
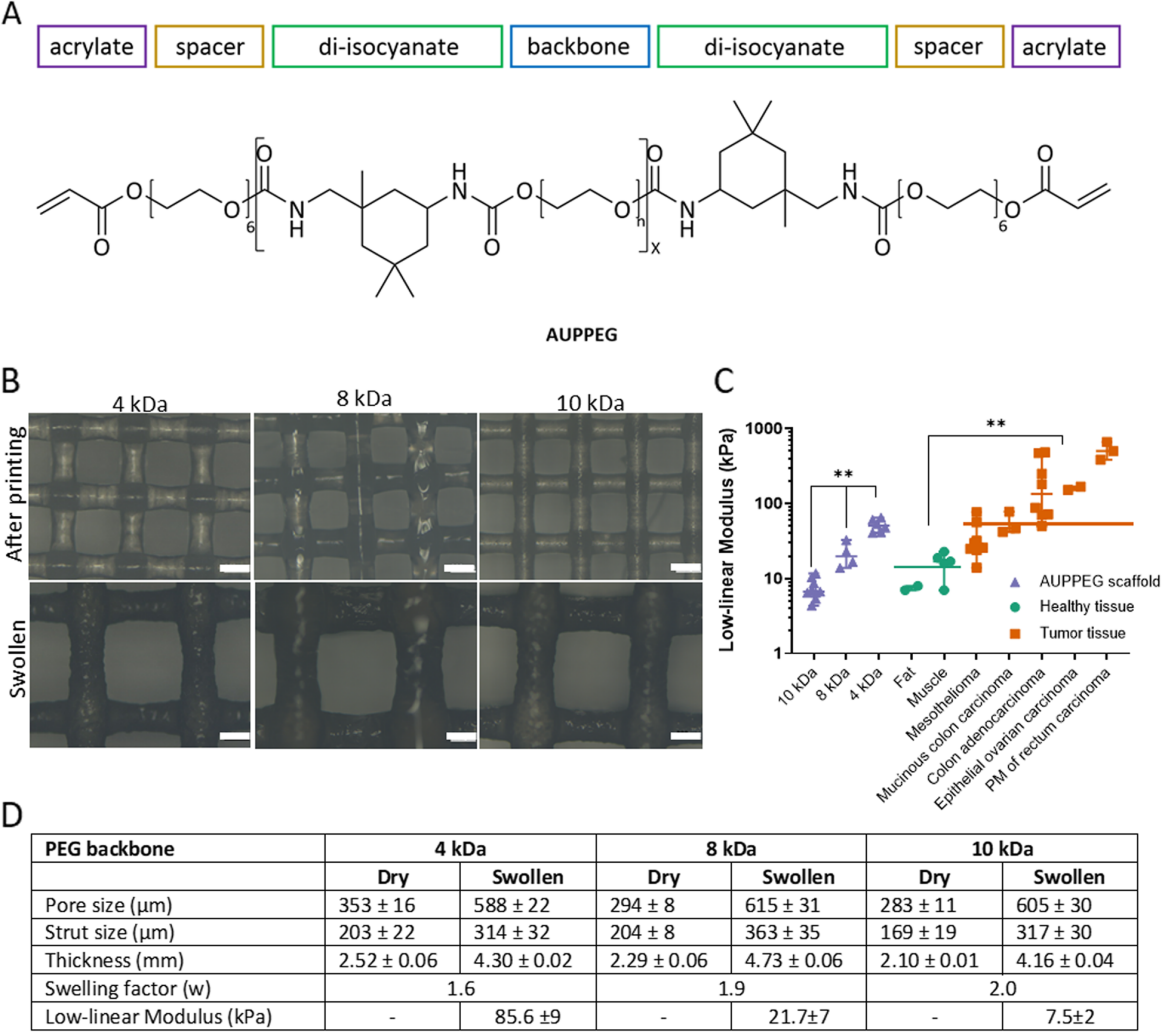
AUPPEG scaffolds. **A** Model compound of an AUPPEG polymer. For the backbone, PEG4k, 8k and 10k were used. **B** Light microscope images of 3D printed AUPPEG scaffolds with 3 different backbone lengths. Printing parameters are adjusted to have similar dimensions in swollen state. Scale bar 200 µm. **C** Indentation measurements of swollen AUPPEG scaffolds with different backbone lengths (*p* < 0.01) and human healthy and tumor tissue. Horizontal line indicates the median stiffness of normal (16 kPa) and tumor tissue (56 kPa) (*p* < 0.01). **D** Table with physical parameters of the AUPPEG scaffolds with the different backbone lengths

To address these goals, we exploited an elastic polymer (acrylate-endcapped urethane-based poly(ethylene glycol) (AUPPEG)), to print scaffolds capable of mimicking tissue-relevant stiffness. AUPPEG polymers combine the interesting complementary properties of PEG and polyurethanes, which are both widely used for their outstanding mechanical characteristics. More specifically, AUPPEG are composed of four building blocks: a backbone, urethane linkers, spacer moieties and crosslinkable terminal groups (Fig. 1A). The urethane groups contribute to the physical hydrogel properties by increasing the toughness while simultaneously acting as a linker between the spacer units and the backbone. The spacer moieties enable efficient crosslinking in the solid state as they provide additional mobility to the terminal acrylate groups. This contributes to excellent shape fidelity for extrusion-based 3D printing. AUPPEG can thus be 3D printed already starting from the solid state/melt. Finally, the crosslinkable groups enable the formation of a covalently crosslinked network upon UV irradiation. AUPPEG materials thus combine the advantageous characteristics of polyurethanes with the biomedical potential of hydrogels. By varying the chemistry of the building blocks, such as the backbone molar mass, the properties of the constructs can be fine-tuned (e.g. controllable stiffness, swelling, …), rendering the AUP hydrogels suitable for versatile biomedical applications [20–23] These unique characteristics of AUPPEG allow to print a scaffold with controllable pore and strut size, and at the same time control the stiffness. Although these scaffolds are relatively soft, they are not fragile and don’t break even after multi-handling, over longer periods of time. This AUPPEG scaffold allows the introduction of single cell suspensions from LGSOC patient-derived early passage cultures of cancer cells and CAF, that through self-assembly, form heterocellular spheroids within the scaffold. This LGSOC AUPPEG scaffold allows long-term (> 40 days) observation and targeted compound evaluation confirmed the durable response of a newly identified drug combination. The drug-response results from this long-term in vitro model are compared with drug responses in an orthotopic LGSOC xenograft mouse model. We conclude that the LGSOC AUPPEG scaffold allows long-term drug evaluation and should be considered as a valid alternative to evaluate drug response in animal models.

## Materials and methods

### Acrylate-endcapped urethane-based poly(ethylene glycol)(AUPPEG) synthesis and structural characterization

AUPPEGs were synthesized as described earlier in [20, 24] via a two-step modification. In brief, dry PEG (Sigma Aldrich) (4, 8 or 10 kg/mol) was first reacted 75 °C with 2 equivalents of isophorone diisocyanate (IPDI, Sigma Aldrich), 500 ppm butylated hydroxytoluene (Innochem GmbH) and 300 ppm bismuth neodecanoate (Shepherd). Next, the endcap Bisomer PEA-6 (Geo Specialty Chemicals) was added dropwise in a 1:2 molar ratio together with 300 ppm of bismuth neodecanoate and the reaction temperature was subsequently increased to 80 °C. The reaction proceeded until no absorption band could be observed at 2270 cm^−1^ using Fourier transform infrared (FTIR) spectroscopy, indicating that the isocyanate groups of IPDI had completely reacted. At the end of the reaction, 500 ppm of both triphenylphosphite (Sigma Aldrich) and phenothiazine (Sigma Aldrich) was added as post-stabilizers. For the synthesis of AUPPEG10K, it is necessary to add butan-2-one at the start of the reaction to lower the viscosity. Butan-2-one was distilled off with water at the end of the reaction. Afterwards, the material was poured into plates and lyophilized using a Christ freeze-dryer alpha 2–4 LSC at − 85 °C and 0.37 mbar.

FTIR spectra ranging from 4000 to 600 cm^−1^ were recorded using a PerkinElmer Frontier FTIR/FIR Spectrometer, equipped with an MKII Golden Gate Single Reflection ATR system, consisting of a diamond crystal and a Sapphire anvil. Proton nuclear magnetic resonance (^1^H NMR) spectra were recorded in deuterated chloroform (CDCl_3_) (Euriso-Top) using a Bruker 400 MHz Avance II Ultrashield and were analyzed using the MestreNova Software. The acrylate content (C_acr_), using dimethyl terephthalate (Sigma Aldrich) as internal NMR standard, and the molar mass (MM) were calculated as reported in [21, 24] (Table S2).

### Scaffold fabrication

#### 3D-printing of AUPPEG scaffolds

**Fig. 2:**
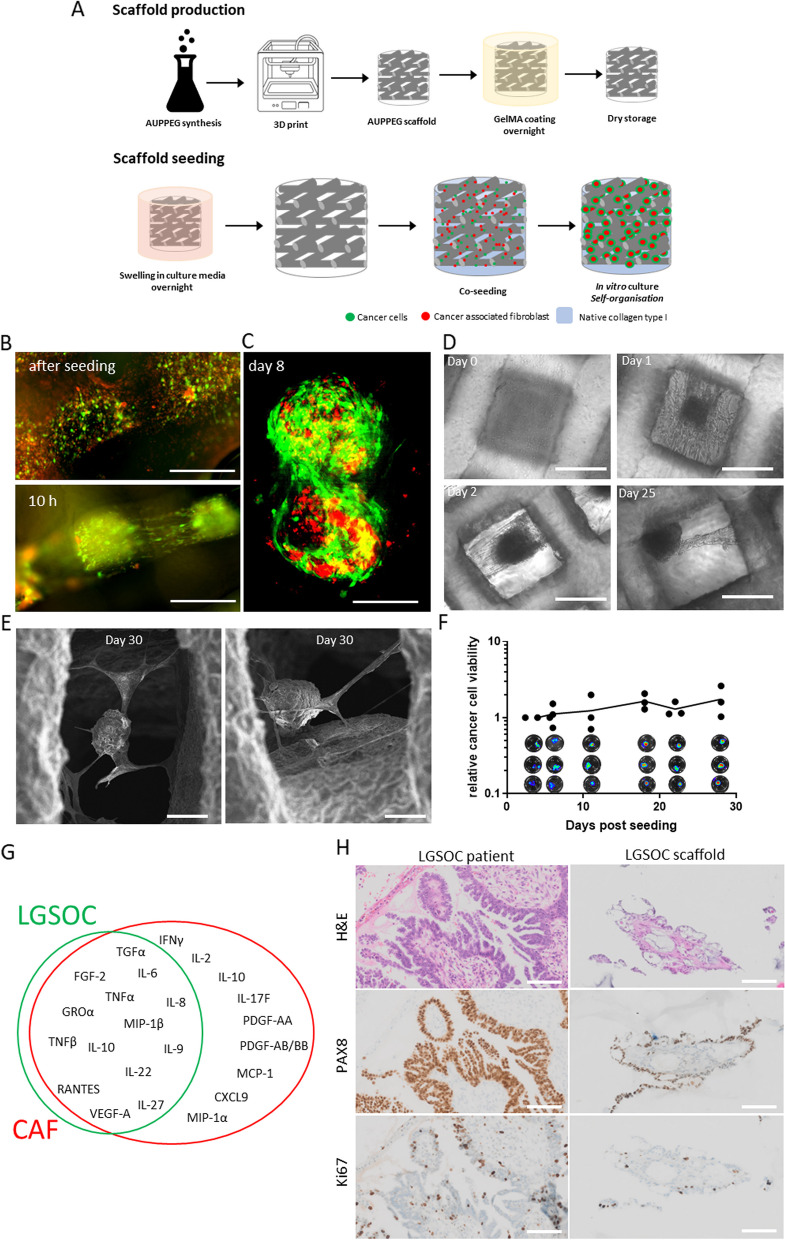
LGSOC scaffold model. **A** Schematic overview of scaffold production and seeding procedure, single cell mixture of cancer cells and CAF suspended in type I collagen solution are added to the scaffold by drip seeding. During in vitro culture cells self-assemble into spheroids, CAF (red) organize at the center surrounded by cancer cells (green). **B** Fluorescent images of PM-LGSOC-01 Luc eGFP (green) and CAF (red) seeded on a AUPPEG8K scaffold, immediately and 10 h after seeding. Round cells (after seeding) become elongated, indication of migration along the collagen fibers, and self-assembly into a spheroid (10 h). Scale bar are 100 µm. **C** Confocal image of the LGSOC scaffold model 1 week post seeding on a AUPPEG8K scaffold. Two spheroids within one pore are visualized; red labeled CAF form the center and LGSOC (green) surround the CAF. Scale bare are 50 µm. **D** Phase contrast images of the LGSOC scaffold model at day 0, 1, 2 and 25 post seeding. Scale bars are 200 µm. **E** SEM images of the LGSOC scaffold model 1 month post seeding. **F** Relative cancer cell viability within the LGSOC scaffold model determined by bioluminescence imaging (BLI). **G** Venn diagram of cytokines identified by Luminex in the secretome of the LGSOC scaffold model. Cytokines are produced by LGSOC cells and/or CAF. **H** H&E, PAX8 (ovarian cancer marker) and Ki67 (proliferation marker) staining of an LGSOC patient sample and the LGSOC scaffold model (> day 30). Note that the early-passage LGSOC cell culture used in the scaffold is established from the patient sample used to evaluate morphology and PAX8 and Ki67 positivity. Scalebar 100 µm

AUPPEG4K, AUPPEG8K and AUPPEG10K scaffolds were manufactured from the melt using the 3D Bioplotter (SysEng Bioscaffolder) (Fig. 2A). The printing parameters were optimized to obtain scaffolds exhiting similar dimensions in swollen state. The optimized parameters are summarized in Table S3 for each material. After printing, the scaffolds were crosslinked using UV-A irradiation from both sides during 40 min (10 mW/cm2, 365 nm).

#### Coating of 3D-printed AUPPEG scaffolds

To improve the bioactivity of the 3D-printed AUPPEG scaffolds, a coating of methacrylated gelatin (GelMA) was applied. To this end, the scaffolds were immersed overnight in a 2 w/v% GelMA solution containing 2 mol% of Irgacure 2959 with respect to the amount of methacrylamide moieties while being protected from light. Afterwards, the scaffolds were punched using a metallic 6 mm puncher, and the gelMA coating was crosslinked using UVA irradiation from both sides for 60 min (10 mW/cm^2^, 365 nm). The GelMA coating was applied to increase cell interaction according [11] (Table S4).

### Physicochemical characterization

*Pore-and strut size:* Scaffolds in dry as well as in swollen state were visualized by a Zeiss Axiotech optical microscope. 4 scaffolds spots were each measured with the ZEN software.

*Swelling factor:* The swelling factor was determined by dividing the pore and strut size in dry state (p_d_ and s_d_) by the size in swollen state (p_s_ and s_s_) (*n* = 4).

$$swelling\;factor=\frac{(\frac{p_d}{p_s}+\frac{s_d}{s_s})}2$$

*Gel fraction:* Crosslinked samples were weighed after lyophilization. The obtained mass was the initial dry weight of the scaffolds (*w*_1_). Next, the samples were equilibrium swollen in double distilled water for 24 h at 37 °C followed by lyophilization. The dry weight after swelling is the mass of the samples after leaching out the non-crosslinked compounds (*w*_2_) (*n* = 4). Using the following formula, the gel fraction was calculated:

$$Gel\;fraction=\frac{w_2}{w_1}.100$$

*Mass swelling ratio:* The samples were first weighed after lyophilization to obtain the initial dry weight mass (*w*_d_). Secondly, the samples were weighted after swelling for 24 h in 37 °C PBS (*w*_s_) (n = 4). The mass swelling ratio was calculated using the following formula:

$$Mass\;swelling\;ratio=\frac{(w_s-w_d)}{w_d}$$

*Stiffness:* As parameter for stiffness the low-linear modulus obtained by indentation was determined, as this allowed comparison between the swollen scaffolds and human tissue samples. The measurements were conducted on the Instron-5944 with Bluehill 3 software (INSTRON, Norwood, MA, USA), a 5 mm flat-end cylindrical indenter connected to a 50 N load cell at a loading rate of 1 mm/s. The low linear modulus was calculated as the slope of the stress to strain curve in the 5% to 10% strain region.

Human samples were obtained from the gastro-intestinal surgery department at Ghent University Hospital from patients undergoing surgery for peritoneal carcinomatosis from various primary tumors (Tables S5, 6). Informed consent was obtained prior to surgery. After surgical resection samples were sliced(4 mm thickness) and taken to the test setup within 1 h.

### Cell culture

Isolation, characterization and culture of patient-derived CAF [25] and low-grade serous ovarian cancer (LGSOC) early passage cultures (PM-LGSOC-01) [26] were previously described. SK-OV-3 is a human ovarian cancer cell line (ATCC number: HTB-77). The PM-LGSOC-01 Luc eGFP and SK-OV-3 Luc eGFP were obtained by retroviral transduction of pLenti6(Blast)-eGFP-V5. CAF, SK-OV-3 and seeded scaffolds were cultured in Dulbecco’s modified Eagle’s medium (DMEM, high glucose) (41965039, ThermoFisher) and LGSOC cells in Eagle's Minimum Essential Medium (EMEM) (10–009-CV, Corning). Both media were supplemented with 10% heat-inactivated fetal bovine serum (FBS) (ATCC-30–2030, LGC Standards), 100 IU/ml penicillin and 100 mg/ml streptomycin (15070063, ThermoFisher). Cells were expanded and maintained as a monolayer at 37 °C in an atmosphere of 10% CO_2_ (CAF, SK-OV-3) and 5% CO_2_ (LGSOC) in air and passaged at 80% confluence.

3D spheroids were formed and cultured in U-shaped, 384-well ultra-low attachment (ULA) plates (cat. no. MS-9384UZ, S-bio) with a suspension of 80 µl cell culture media with 2 × 10^3^ cells per well for monocultures and 1 × 10^3^ LGSOC combined with 4 × 10^3^ CAF, with or without 50 µg/ml type I collagen for co-cultures; for mono-cultures the 2D growth medium was used, for co-cultures DMEM HG was used. Cells were tested monthly for mycoplasma contamination using the Mycoalert Mycoplasma Detection Kit (LT07-318, Lonza).

### Cell seeding procedure onto scaffolds

Scaffolds were seeded with a combination of CAF and cancer cells, as a single cells (Fig. 2A) (PM-LGSOC-01 Luc eGFP or SK-OV-3 Luc eGFP). Swollen scaffolds obtained after 1 h incubation in culture media 37 °C were seeded by dripping 0.2 ml of single cell suspension in collagen type 1 solution on top of the scaffold. 1 ml of 2 mg/ml type I collagen suspension contains: 557 µl collagen type I (3.5 mg/ml, sc-136157, SantaCruz), 79 µl CMF-HBSS), 72 µl MEM10X, 72 µl NaHCO3, 20 µl NaOH 1M and 200 µl cell suspension (5 × 10^6^ cancer cells and 20 × 10^6^ CAF). The scaffolds were inverted 30 min past seeding and left to settle for another 30 min. During the seeding procedure scaffolds were placed in a 24 well plate on a heating plate (37 °C) followed by culturing in an incubator at 10% CO_2,_ 37 °C in 1.5 ml of culture medium_,_ medium is refreshed twice a week. If scaffolds were used for fluorescent or confocal imaging, CAF were stained with IncuCyte® Nuclight Rapid Red Cell Labeling (4717, Sartorius).

### Microscopy

Optical microscopic images of printed scaffolds were obtained using an Axiotech microscope (Zeiss) using top down illumination in combination with an Axiocam for image acquisition. Raw images were processed using the ZEN software package. Seeded scaffolds were imaged with a Leica DMI3000B phase contrast microscope running the LAS4.1 software package. Epi-fluorescence images were obtained on Incucyte® ZOOM (Sartorius). Confocal microscopy images were recorded on a Leica DMI 6000 inverted microscope coupled to an Andor D8D2 spinning disc system and an Andor Zyla 5.5 CMOS camera. A 10x (NA 0.4) objective was used and Z-stacks were recorded with 1 µm spacing. Images were processed in the Image J software package. Scanning electron microscopy (SEM images are acquired at an acceleration voltage of 7 kV using an JSM-6010PLUS/LV SEM device (JEOL). Before imaging, the scaffolds were fixed in 2.5% glutaraldehyde in cacodylate buffer, snap frozen by liquid nitrogen, dehydrated by lyophilization and Au-coated with a JFC-1300 Auto Fine Coater (JEOL) to avoid charge effects. Haematoxylin & eosine (H&E) and immunohistochemistry (IHC) sections were imaged on a Olympus BX51 virtual microscope, with motorized stage BX-REMCB and controlled by VS-ASW software package (Olympus).

### In vitro and in vivo cancer cell viability

Cancer cell viability in seeded scaffolds and in vivo experiments was determined by bioluminescence imaging (BLI). BLI was performed with the IVIS Lumina II (PerkinElmer) and quantified with Live images 4.3 (PerkinElmer). In vitro bioluminescent images were immediately acquired after placing the scaffold in 1 ml culture medium containing 150 µg D-Luciferin firefly (PerkinElmer). Scaffolds were placed in fresh medium after imaging. In vivo image acquisition was performed 15 min after intraperitoneal injection of D-Luciferin firefly (200 µl of 15 mg/ml solution, PerkinElmer). The image acquisition parameters were: binning factor medium, F/Stop: 1 and exposure times were set automatically.

### Histology

Scaffold and tumor samples were fixed in 3% buffered formalin for 1 h and immersed in increasing concentrations of alcohol to dehydrate the tissues prior to paraffin embedding. Xylene and Ultraclear (J.T. Baker) were used as clearing and deparaffinizing agents for tumor samples and scaffolds respectively. Sections were stained using a standard H&E and immunohistochemistry protocol applied in clinical pathology. For antibody list see Table S7. Images were quantified with Image J.

### Cytokine analysis—Luminex

Collected media from LGSOC scaffolds (day 54–58), 3D CAF-spheroids (day 2–6) and 2D LGSOC cultures (day 2–6) were passed through a 0.2 µm filter and processed with the Human Cytokine Array / Chemokine Array 48-Plex (HD48) by Eve Technologies. Background values where determent on culture media.

### In vivo* experiments*

**Fig. 3:**
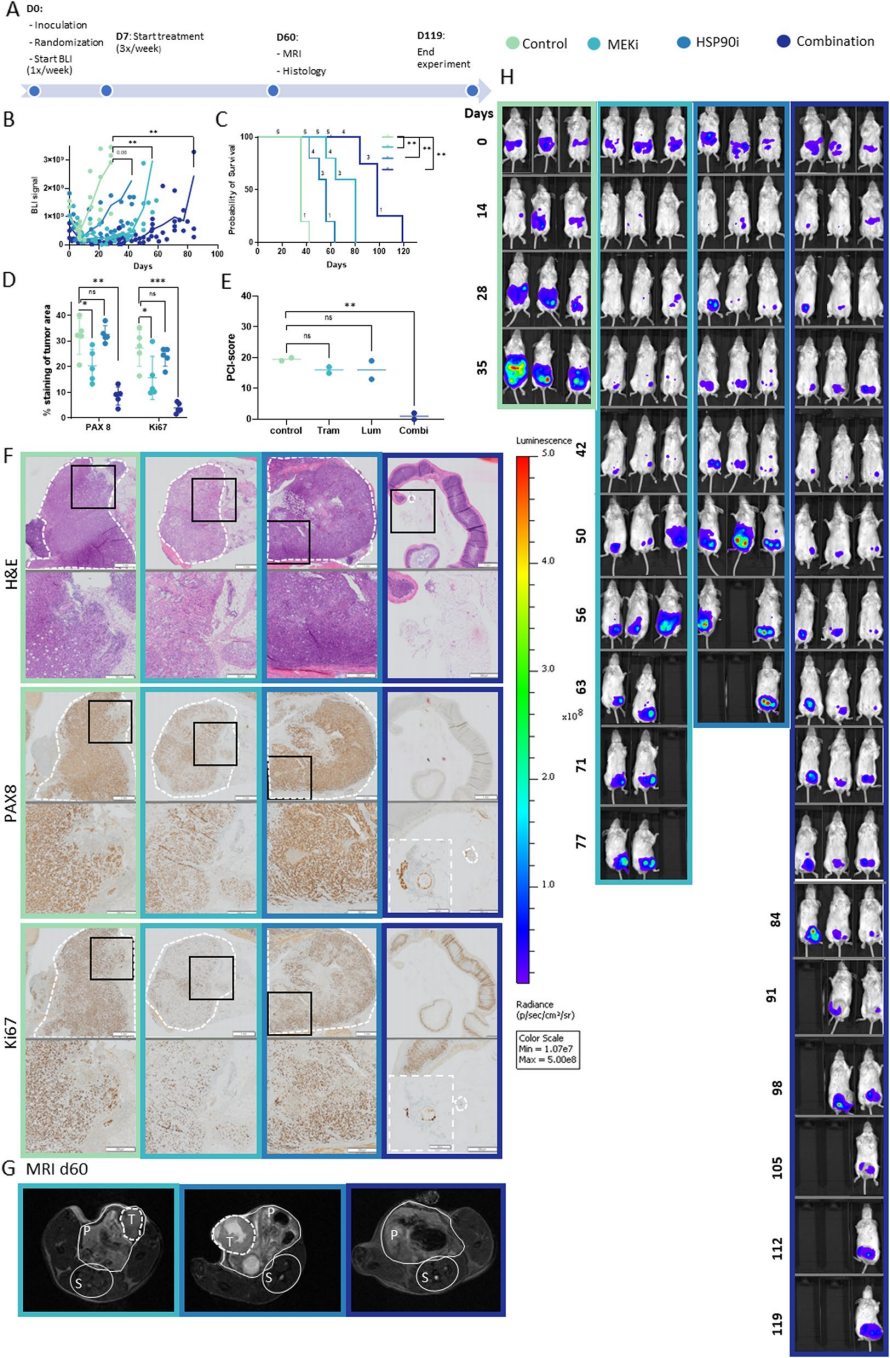
MEKi/HSP90i treatment of LGSOC intraperitoneal LGSOC mouse model. Treatment groups are indicated by color code for the entire figure. **A** Timeline of the mice experiment. At day 0 (D0) mice are inoculated with PM-LGSOC-01 Luc eGFP and randomized according to their BLI signal. At day 7 (D7) MEKi/HSP90i treatment is started and continued 3 times a week till humane endpoint is reached and mice are euthanized. At day 60 (D60) MRI is performed on one mouse per group, except for the control group, as all control mice reached the endpoint before day 60. At the same timepoint two mice per group are euthanized for intermediate histology analysis; for the control group, mice at the endpoint are used. **B** Weekly BLI results of individual mice are indicated in a graph with colored dots representing individual mice, and a continuous colored line indicating the average BLI. **C** Kaplan–Meier survival curve based on the humane endpoint. Numbers in the curve indicate the number of animals at risk. **D** Quantification of immune histology staining’s performed on five tumor regions of the intermediate euthanized mice. **E** Peritoneal carcinomatosis index (PCI)-score of the intermediate euthanized mice. **F** (Immuno)histology of intermediate euthanized mice. In each panel, the bottom image is a larger magnification of an area indicated by a black square in the upper panel. In the combination treatment, an inset in the larger magnification indicates an additional enlargement of a tumor area indicated by a white dotted line. Scale bars are respectively 1 mm, 500 µm and 100 µm. **G** MRI scans at day 60, dotted line indicates the tumor (T). In the combination treatment no tumor could be detected at day 60. The spinal cord (S) and peritoneal cavity (P) are indicated. **H** BLI images of 3 individual and representative mice monitored up to 119 days. *p < 0.05, **p < 0.01 and ***p < 0.001

Animal experiments were carried out in accordance with the regulatory guidelines of the Ethics Committee of the Ghent University Hospital. 28 female SCID/Beige (C.B-17/IcrHsd-PrkdcscidLystbg-J) mice of 5 weeks old (Envigo) were intraperitoneally injected with 1 × 10^6^ PM-LGSOC-01 Luc eGFP (1:1 serum free EMEM medium:Matrigel (Corning)). One week post inoculation mice were randomized into four groups with equal average bioluminescent signal and treatment was started. Mice were treated 3 times a week. Trametinib (0.3 mg/kg) is given through oral gavage with vehicle (0.5% methylcellulose and 0.2% Tween-80 in demi-water). Luminespib (5 mg/kg) treatment was intraperitoneally injected, the combination treatment included Trametinib oral gavage directly followed by intraperitoneal Luminespib injection. The control group received a solvent control treatment. Follow-up by BLI was performed weekly. At day 60, one mouse per group was imaged by Magnetic Resonance Imaging (MRI) using a T2-weighed sequence (TurboRARE, TR = 3661ms, TE = 37.1ms, 30 slices with voxel size 120 µm × 120 µm x 600µm, 6′20″ acquisition time) (Pharmascan 70/16, Bruker BioSpin) and two mice per group were sacrificed for intermediate evaluation by Peritoneal Carcinomatosis Index (PCI score) and histology. The PCI score is a descriptive score of the spread of tumor nodules across the peritoneal cavity and was performed according to Derrien et al*.* Scores from 0 to 3 were defined for each region (0): no macroscopic tumor; (1): lesion from 1 to 2 mm, 1 to 2 sites; (2): lesion from 2 to 4 mm, 1 to 2 sites; and (3): lesion over 4 mm or more, on 13 sites. The total PCI score is the sum of the score for each region and ranged from 0 to 39 [27]. The 20 remaining mice were continuously evaluated for survival analysis and end-point measurements. Humane endpoint was set at tumor load (BLI > 1 × 10^10^) or weight loss (> 20%). For time line see Fig. 3A.

### Scaffold treatment

LGSOC scaffolds were treated 3 weeks past seeding by refreshing the culture medium two times a week with media containing treatment; control (0.1% dimethylsulfoxide (DMSO)), Trametinib (1 nM Trametinib, C988930 Bioconnect), Luminespib (10 nM Luminespib, ORB154741, Bioconnect) and combination (1 nM Trametinib and 10 nM Luminespib). 46 days after the first treatment, culture media was collected after 96 h past media refreshment, passed through a 0.2 µm filter and stored at -20 °C for Luminex and glucose and lactate concentration measurements.

### Glucose and lactate concentration measurements

Glucose and lactate concentrations in culture medium of treated scaffolds were measured using enzymatic assays involving bioluminescent NADH detection technology and a selective dehydrogenase (Glucose-Glo J6021 and Lactate-Glo J5021 assay; Promega, Madison WI, USA). For the glucose-glo assay, collected media was diluted 1:500 in PBSD^-^, for the lactate-glo assay a dilution of 1:100 in PBSD^-^ was used. Controls (medium without cells) were diluted correspondingly. 50 µl of sample was pipetted into white micro 96-well plates (236108, ThermoFisher) and an equal volume of assay reagent was added. The contents were mixed for 30 s on an orbital shaker while shielded from light. Luminescence readout was performed after 1 h incubation at room temperature. Glucose consumption and lactate production was calculated by taking into account the glucose or lactate concentrations of medium without cells.

### Statistics

Equivalence tests were performed in Jamovi [28] by TOST with 10% deviation set as acceptable. All other statistical analyses were performed with GraphPad Prism 8. Growth curve analysis, both mice and scaffold model were analyzed by “mixed effect models”, *p*-values of treatment effect independent from time were reported. Survival analysis was performed with Log-rank (Mantel-Cox) test. Correlation was done with the Spearman correlation test. Comparisons of different groups (> 2) were determined with a Kruskal–Wallis test with Dunn’s correction, (comparing stiffness, quantification of PAX8 and Ki67, PCI-scores). When comparing only two groups, Mann–Whitney U was implemented (comparing glucose consumption, lactate production and lactate/glucose ratio). Alfa of 0.05 is considered significant.

## Results

### Development of a tissue stiffness-relevant AUPPEG scaffold model

We previously presented a poly-lactic acid (PLA)-based scaffold which mimics the 3D cellular organization of peritoneal metastases from HGSOC [11]. However, the biomechanical properties of the PLA scaffolds did not match those of an in vivo tumor, since PLA is a 100 to a 1000 fold stiffer than tumor tissue [11], (Fig. 1C). In addition, 3D heterocellular spheroid formation and TME reconstitution in the scaffolds needed 4 weeks of culturing, excluding the possibility for drug treatments shortly after cell seeding onto the scaffold (Fig. S1) [11]. To match tumor relevant stiffness, we replaced PLA by an elastic AUPPEG polymer that, upon printing, will maintain a stable scaffold with open pores to allow cell seeding and preserve its structure during multiple handling procedures. By changing the length of the PEG backbone in the AUPPEG polymer, the biophysical properties (i.e. swelling degree, mechanical properties) can be adapted according to the biological needs [20]. AUPPEGs with PEG backbones of variable length (10, 8 and 4 kDa) were printed and the controlled architecture was analyzed through microscopy. Pores and struts had similar dimensions in swollen state (equivalence TOST *p* < 0.01) (Fig. 1B, D). The backbone length was positively correlated with the swelling factor and negatively with the stiffness. A longer backbone caused more swelling, resulting in a softer scaffold (Fig. 1C-D). The stiffness of these scaffolds was compared to that of human healthy and fresh tumor tissue obtained from the operation room (Tables S5, S6). The low-linear modulus measures the resistance to indentation and higher pressures damage the tissue and alters the resistance [29]. Peritoneal tumor samples from variable sources had a higher stiffness compared to non-tumor peritoneal adipose and muscle tissue. Interestingly, among the different tumor tissues analyzed, the highest stiffness was observed in advanced metastasis and the lowest in a mucinous form of colon cancer characterized by abundant extracellular mucin which accounts for at least 50% of the tumor volume. AUPPEG 8 kDa scaffolds mimicked the stiffness of non-tumor tissue, while AUPPEG 4 kDa mimicked the stiffness of tumor samples from variable sources (Fig. 1C).

### TME components are essential in 3D culture of LGSOC

Although we understand increasingly better the molecular features of LGSOC, we also need insights into its microenvironment for proper understanding of therapeutic response and tumor modeling. Representative images of LGSOC tumors revealed the presence of alfa-smooth muscle actin positive CAF surrounded by tubular structures that stained positive for the ovarian cancer cell marker PAX8 (Fig. S2A). CAF contributed to tumor characteristics by matrix production and cytokine/growth factor secretion. The intimate interaction between CAF and LGSOC cells inspired us to recreate this interaction in vitro by combining patient-derived early passage LGSOC cells and CAF. Suspensions of both cell types in presence of type I collagen formed a compact heterocellular spheroid. In the absence of TME components, the compact heterocellular spheroid structure was lost. In addition, H&E analysis revealed a core of densely packed CAF surrounded by LGSOC cells (Fig. S2B-C). Although compact heterocellular spheroids are formed that reconstitute the LGSOC TME within 48 h and which allow drug evaluation, it is well known that these spheroids do not allow long-term evaluation in ULA plates due to disintegration of spheroid structures and loss of spheroids by medium refreshments [30].

High grade tumors and metastasis typically show fast proliferating cancer cells [18], fibrotic ECM heterogeneity [31] and higher stiffness (Fig. 1C). LGSOC tumors are characterized by well differentiated micro papillae and small nests of cancer cells that lack characteristics such as fast proliferation and fibrotic ECM [18]. Therefore, we seeded LGSOC cells on scaffolds with an 8 kDa PEG backbone showing a stiffness similar to healthy tissue (Fig. 1C). This created a clinically relevant 3D model for LGSOC that allows necessary medium refreshments and long term follow-up. Making use of red-labeled CAF (Nuclight Rapid red) and green-labeled LGSOC cells (eGFP), we observed a mix of single cells upon seeding that self-assembled into compact heterocellular spheroids within 48 h. Initially, single cells showed a rounded morphotype that evolved into elongated cells. Video microscopy revealed the migration of LGSOC cells along the collagen fibers and confocal microscopy identified a core of CAF surrounded by LGSOC cells in the spheroids (Fig. 2B-D). Scanning electron microscopy and phase-contrast microscopy revealed the prolonged presence of compact spheroids attached to the AUPPEG 8 kDa scaffolds by collagen fibers (Fig. 2D-E). Interestingly, microscopy revealed that the collagen hydrogel in which the cells were seeded contracts over time. This is most likely through CAF-mediated contractile activity, resulting in the creation of voids within the scaffolds, which in turn allow nutrient exchange and drug penetration, further ensuring the long-term evaluation of the LGSOC scaffolds. The luciferase reporter in LGSOC cells revealed the continuous and exponentially increasing viability of the heterocellular spheroids up to one month of culturing (Fig. 2F). Thus, biweekly medium refreshments during four weeks did not impact spheroid organization and viability (Fig. 2D-F). Further functional evidence of LGSOC TME reconstitution in the scaffold was provided by cytokine analysis of secretomes. Growth stimulating (PDGF-AA, PDGF-AB/BB, TGFα, FGF-2 and VEGF-A) and immune engaging (interleukins, chemokines and TNFβ and IFNγ) cytokines were released into the supernatant. While 40% of the cytokines were CAF specific, the majority were contributed by both LGSOC cells and CAF (Fig. 3G).

LGSOC tumors have typically a differentiated phenotype characterized by duct formation visible as tubes on histological sections. Interestingly, this histological growth pattern was reproduced in the AUPPEG 8 kDa scaffolds and was maintained during long-term culture. The ovarian cancer cell marker PAX8 remained present after long-term culture. The nuclear proliferation antigen, Ki67, showed positivity in the scaffolds indicating actively proliferating cells. Interestingly, not all cells showed positivity, indicating slower growth, a typical characteristic of LGSOC (Fig. 2H).

The fast (48 h) and durable (at least 4 weeks) mimicry of LGSOC TME in AUPPEG 8 kDa scaffolds allows long-term drug evaluation shortly after scaffold seeding.

### Therapy screening for LGSOC

To evaluate long-term effects of drug treatments on LGSOC AUPPEG 8 kDa scaffolds, we first identified potentially interesting single and combination drug treatments. An ATP viability screening identified 6 compounds with favorable dose–response activity (Fig. S3A). Two compounds target complementary pathways driving cell proliferation and survival and, when combined, may thus potentially strengthen each other’s efficacy. Trametinib is a potent MEKi, targeting both MEK1 and MEK2 isoforms, with clinical relevant activity in LGSOC, while the Heat Shock Protein (HSP)90i Luminespib targets the chaperone HSP90 resulting in unproper folding of client proteins such as the prosurvival protein AKT (Fig. S3B). The impact on cell viability by pharmacologic targeting of MEK and HSP90 was confirmed by transfection of siRNAs targeting MEK1 + MEK2 or HSP90 (Fig. S3D). Interestingly, while MEK silencing revealed a complete absence of ERK phosphorylation, a marked increase in compensatory AKT phosphorylation and its downstream target mTOR was observed. In contrast, HSP90 silencing had no impact on ERK activity levels but markedly reduced AKT and mTOR phosphorylation (Fig. S3C). Further mechanistic rationale to combine MEKi with HSP90i came from proteomic experiments. Single treatments of LGSOC cells with either MEKi or HSP90i revealed effects on complementary pathways with a majority of changed proteins in the MEKi group related to cell cycle and kinase signaling, while the HSP90i mainly affected proteins related to stress response and protein folding. Shared affected proteins by single compound treatments were implicated in the TME, including cell matrix interaction (Fig. S3E). Thus, favorable dose–response viability results, genetic perturbance and proteomic profiles suggest that single and combination treatment of MEKi and HSP90i are of interest to evaluate in long-term in vivo and in vitro assays of LGSOC.

### MEKi and HSP90i treatment evaluation in an intraperitoneal LGSOC mouse model

We first tested the in vivo efficacy of single and combined compound treatment in a LGSOC peritoneal metastasis model evaluated by luminescence monitoring and end-point MRI, macroscopic and microscopic evaluation (Fig. 3A). Vehicle-treated mice showed peritoneal metastasis formation and all mice reached the humane endpoint between 35 and 42 days post inoculation. As expected from clinical data and biochemical and functional assays, the MEKi significantly delayed tumor formation and almost doubled the survival time. Interestingly, detailed inspection of the luminescence curves showed a low but stable luminescence signal until day 40, after which the tumor started to grow at the same rate as in the control group as evidenced by equal slopes between both curves (vehicle m = 1.2e8 vs MEK m = 1.7e8). These results strongly suggest an initial response followed by the appearance of drug resistance and fast tumor growth. Animal death started at day 56 and all animals succumbed by day 80. HSP90i-treated mice showed no pause in tumor growth, as was observed for MEKi, but showed slower tumor growth as evidenced by a difference in slope (m = 5.9e7) of the curve compared to vehicle treatment (m = 1.2e8). Combined MEKi and HSP90i treatment showed the most significant delayed tumor formation and increased animal survival of all treatments. First animal death in the combined treatment group started 4 days after the last animal died in the MEKi group, resulting in an overall 50% increase in survival compared to MEKi only and an almost triple increase in survival time compared to vehicle treatment. Luminescence monitoring revealed three phases; an initial low, but stable signal up to day 50 as is observed for single MEKi treatment, followed by a second phase up to day 80 with a slow, but steady increase in luminescence signal (m = 2.95e7) ending into a last phase marked by a steep increase in luminescence over a 10-day time frame (m = 2.3e8) (Fig. 3B, H). At day 60, a time-point when all animals succumbed in the vehicle-treated group, we performed MRI (Fig. 3G) and identified the largest tumor in the only remaining animal of the HSP90i-treated group. Differences in color intensity may mark large necrotic areas as was also evidenced by histological analysis (Fig. 3D, F). Although luminescence signals started to increase in the combination treatment, no tumor was observed yet by MRI. The steep increase in luminescent signals in the MEKi group at day 60 was also evidenced by identification of tumor regions on MRI (Fig. 3G). Additionally, at day 60, two mice per group were euthanized for intermittent analysis; for the control group, previously succumbed endpoint mice were used. PCI (Peritoneal Cancer Index) scores (Fig. 3E), an indication for tumor spread in the abdominal cavity, were in line with the growth, survival and MRI data (Fig. 3B, C, E and H). Immunohistological quantification was expressed as a percentage of tumor area, a score independent of tumor size. The control (endpoint) and HSP90i group (near endpoint) had a similar positivity for PAX8 and Ki67. It must be noted that at this advanced stage of peritoneal metastasis in the animal model, the vast majority of cancer cells showed Ki67 positivity (Fig. 3D, F). This observation is in agreement with the steep slope of the luminescent growth signals (Fig. 3B). While a low Ki67 positivity index is a diagnostic feature for LGSOC, patients typically have slow progressing tumors. MEKi treatment resulted in lower levels of PAX8 and Ki67, an effect which is even more pronounced for the combination treatment (Fig. 3D, F). Although LGSOC was characterized by epithelial cells organized into papillae surrounded by stroma, at this end stage, this spatial organization was lost except for the combination group in which not only significantly delayed tumor growth was observed but also a reorganized tumor structure as evidenced by presence of larger and more differentiated ductal structures.

### MEKi and HSP90i evaluation in LGSOC scaffold model allowing long-term treatment

**Fig. 4:**
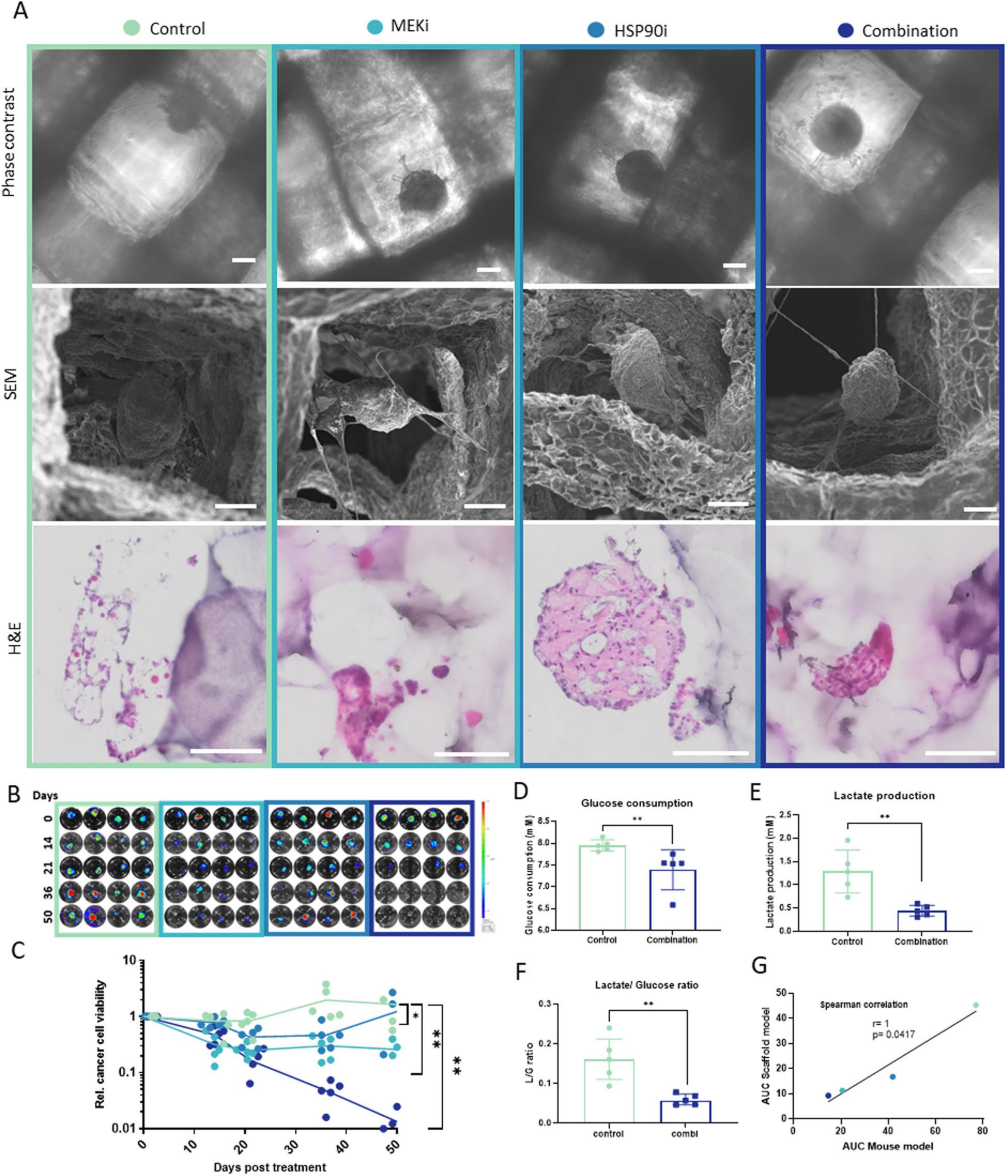
MEKi/HSP90i treatment of LGSOC scaffold model. Treatment group is indicated by color code for the entire figure. **A** Images of treated LGSOC scaffold model at day 50; phase-contrast, scanning electron microscopy (SEM) and Hematoxylin & Eosin (H&E). Scale bars 100 µm. **B** Relative cancer cell viability within the treated LGSOC scaffold model determined by BLI. **C** Total (cancer cells and CAF) glucose consumption (**D**) and lactate production (**E**) and lactate/ glucose ratio (**F**) of control- and combination-treated LGSOC scaffolds from day 47 till 50. **G** Correlation between treated LGSOC scaffolds and mouse model, based on the AUC of the growth curves. *p < 0.05, **p < 0.01 and ***p < 0.001

We next evaluated whether the in vivo results could be reproduced in the LGSOC scaffold model in which we evaluated drug treatments up to 7 weeks by luminescence monitoring. After 3 weeks, all three treatment groups showed an initial response; cancer cell viability dropped with 75%, 60% and 85% respectively in the MEKi, HSP90i and combination treatment group. In the next two weeks, cancer cell viability remained stable in the single treatment groups and dropped further in the combination group. Intriguingly, in the last phase of the evaluation we observed a rise in the viability of the HSP90i treatment group, a stable signal in the MEKi treatment group and a further drop in the combination treatment close to the detection limit of the luminescence assay (Fig. 4B, C). The limited viability signals are not due to spheroid loss as the LGSOC structure remained intact within the scaffold (Fig. 4A). By none of the therapies, nor the prolonged culturing procedure the spheroid structure was affected as evaluated by phase contrast microscopy and SEM. Since the cancer cell specific luminescent signal was lost in the combination treatment group, the presence of an intact spheroid most probably indicates a continued presence of CAF in which integrity was not visually affected by the treatment. Cancer cells typically maintain their energy demands by aerobic glycolysis, which can be evaluated by measuring glucose and lactate levels in the scaffold supernatant. Both glucose consumption and lactate production were significantly reduced by the combination treatment (Fig. 4D-E). Furthermore, the combination treatment reduced aerobic glycolysis since the ratio of glucose uptake to lactate secretion (L/G ratio) was more than two-fold lower compared to the control treatment (L/G ratio control = 0.16; combi = 0.059). (Fig. 4D-F)). Most interestingly, comparing the luminescent signals of the animal experiment and the scaffold experiment revealed a strong positive correlation coefficient (Spearman *r* = 1, *p* = 0.0417). Consequently, the results from the LGSOC AUPPEG scaffold were indicative of the study outcome in the animal experiments (Fig. 4G).

## Discussion

Due to ethical, biomedical and financial concerns, the need for disease relevant in vitro cultures that allow long-term evaluation is increasingly recognized. To meet this need, we have developed AUPPEG scaffolds to culture heterocellular spheroids of cancer cells and elements of the TME. In addition, LGSOC is a poorly studied tumor type that has limited availability of models and that is in high need for novel treatment options. Creating a clinically relevant in vitro model for an unmet medical need such as LGSOC requires insights into the in vivo characteristics that influence cellular behavior and therapy response. We have thrived to recreate the biological context of a LGSOC tumor with regard to stiffness, heterocellularity and inclusion of ECM matrix compounds. Comparing stiffness of biological tissue and synthetic material can be challenging. Biological tissue has a different compression profile compared to synthetic material [29]. Furthermore, stiffness can be measured by a range of different techniques and settings, which will all result in slightly different values [32]. To overcome this issue, we performed indentation measurements on fresh human samples on the same device, with the same settings as our synthetic scaffolds. We compared the low-linear modulus, indentation between 5 and 10%, as up to this point, strain to stress profiles were comparable between biological and synthetic material. Beyond 10% indentation the integrity of biological samples was damaged and the strain–stress curve changed accordingly. The length of the AUPPEG backbone determines the stiffness of the scaffold and adjusting the backbone allowed us to mimic the ranges of stiffness observed both in healthy as well as diseased tissue (Fig. 1B-D) [33]. It is widely accepted that matrix stiffness influences (cancer)cell behavior [34–37]. Indeed, compared to a previously published PLA scaffold the speed of self-organization into 3D spheroids upon cell seeding into AUPPEG scaffolds increased dramatically. This effect was independent on the cell type since SK-OV-3 cells, model for HGSOC, as used in the PLA scaffold similarly showed faster self-organization in the AUPPEG scaffold (Fig. S1). This efficient self-organizing aspect allows to initiate drug treatments maximally 48 h after cell seeding.

By seeding a single cell suspension of cancer cells and CAFs in type I collagen in tissue-stiffness relevant scaffolds we recreated important elements of the TME of LGSOC [38]. In addition, the presence of CAF provides a protective environment during scaffold seeding, a mechanism that is also used by disseminating cancer cells in vivo. Heterocellular spheroids of CAF and cancer cells are present in ascites of ovarian cancer patients. Upon suspension in ascites fluid, CAF help the disseminated cancer cells to survive in the peritoneal cavity before they are able to adhere to the mesothelial cells lining the peritoneum to form a metastatic implant [5]. Indeed, when we mimicked this suspension phenotype in vitro we observed that CAF are essential for 3D spheroid culture of the patient-derived early passage LGSOC cells both in ULA (Fig. S2) and the AUPPEG 8 kDa scaffold (Fig. 2). A second element is that in LGSOC tumors epithelial cells surround CAF clusters and that these epithelial cells are typically organized in tubular structures as observed in histological slides of patient tumors (Fig. 2H). Interestingly, both in ULA and scaffolds we reproduced this typical LGSOC spatial organization in the presence of CAF. In addition, when comparing the spatial organization and proliferation rate (Ki67) of LGSOC/CAF in the scaffold, the mouse model and patient tumor (Figs. 2H and 3F), our data demonstrate that the scaffold model recapitulated the patient’s tumor better than the mouse model. The current in vitro experiments with the scaffold model do not study angiogenesis and immune responses. Further research is needed whether inclusion of endothelial cells or immune cell types in the scaffold would influence drug response or will allow the inclusion of anti-angiogenesis or immune modulating drugs in the screening procedure. Scaffolds models will not completely replace the need for animal testing, however we believe it has the potential to improve pre-clinical evaluation and further limit the number of animals needed. At the current stage both bovine serum and rat tail collagen are necessary for cell growth and organization in the scaffold, further research may lead to a xeno-free design of the LGSOC scaffold model. But why do we need a scaffold structure and not just heterocellular spheroids in ULA suspension culture? Culturing cells in a scaffold structure has the important advantage of facilitating long-term in vitro 3D culture without the need for cell dissociation and without issues of culture medium refreshments which disturb spheroid integrity and causes loss of spheroids during the process. An additional advantage is the simplicity to take intermediate samples from the culture supernatant. At each culture medium refreshment, the replaced medium can be used for intermediate analyses like cytokines, metabolites and nutrients (Figs. 2G and 4D-F). Both spheroid and organoid models are used as tissue-relevant screening tools [39, 40]. However, long-term treatments to study late stage effects such as durable drug efficacy are currently not performed with classical spheroid or organoid models. Although efforts are ongoing to create scaffolds with tissue-relevant stiffness and presence of TME elements that allow long-term evaluation, few of them have been evaluated for drug efficacy (Table S1). One study evaluates drug response in a glioblastoma scaffold model combining mechanical properties and endothelial cells as stromal element [15]. Glioblastoma and LGSOC are very different tumor types with very different TME interactions. While endothelial cell targeting is necessary to evaluate potential therapies for glioblastoma [41], CAFs are of high importance in ovarian cancer since they play a pronounced role in therapy resistance [42] and across carcinoma types CAF abundance results in a poor survival rates [4].All these elements demonstrate that in vitro models need to be fine-tuned to the characteristics of the in vivo tumor. Considering all these elements, the presented LGSOC AUPPEG scaffold model is clinically relevant and has unique characteristics needed for long-term drug evaluation [10, 43].

LGSOC is a rare and often lethal cancer when diagnosed at an advanced stage. Research models have been challenging to develop. We previously established and characterized an early-passage patient-derived LGSOC cell culture that reflects the molecular make-up of *KRAS* mutated LGSOC tumor [26]. Of the few LGSOC cell cultures available worldwide, our culture model forms peritoneal metastasis upon intraperitoneal injection in mice reflecting advanced LGSOC disease. MEKi showed promising results in a recent phase II/III trial [19, 44] and showed activity in our in vivo model (Fig. 3) However, as can be expected from other tumor types where MEKi are used [44], MEKi sensitivity decreases resulting in fast progressing MEKi resistant tumors (Fig. 3B, C, H). The development of acquired resistance is inevitable due to the signaling pathway rewiring. Drug resistance is one of the most pressing problems in treating cancer patients. A 38-compound screen revealed an HSP90i with favorable dose–response effectiveness on early passage LGSOC cell cultures. Interestingly, It has been demonstrated that MEKi combined with HSP90i show beneficial effects in MAPK pathway activated glioblastoma [44]. HSP90 is a chaperone that is crucial for the stability and function of many proteins and essential for cell survival [45]. Importantly, HSP90 client proteins are associated with various oncogenic proteins including proteins in the MAPK and PI3K/AKT pathway [44]. Indeed, dedicated Western blotting experiments and unbiased proteomic data revealed complementary targeting by single treatments of LGSOC cell cultures with either MEKi or HSP90i, pointing to a rationale for combining both. Our in vivo model pointed to more durable efficacy of the drug combination compared to single treatment leading to significantly longer survival benefit. Although the treatment results within this study are not the main outcome of this research, we strongly warrant further translational and clinical studies to verify this strategy in the appropriate clinical setting.

## Conclusions

Today, the gold standard for long-term drug evaluation are tumor transplanted animal models. However, replacement by in vitro models is the ultimate goal for laboratory animal-based research. Interestingly, results of the single and combined drug evaluation in the long-term LGSOC AUPPEG scaffold model correlated with results obtained using the in vivo model, suggesting that the scaffold model predicts results in the animal model. Although the use of this long-term AUPPEG scaffold as the default option in LGSOC science is too premature, we would like to highlight the need for further objective, unprejudiced monitoring, and robust performance indicators of in vitro approaches.

### Supplementary Information

**Additional file 1: Fig. S1.** Peritoneal metastasis scaffold model with SK-OV-3. **Fig. S2.** CAF in LGSOC tumors. **Fig. S3.** LGSOC 2D monocultures.

**Additional file 2. **Supplementary materials and methods [46–49].

**Additional file 3: Table S1.** Overview of long term (> 4 weeks) in vitro 3D cancer models [50–67]. **Table S2.** Overview of the synthesized AUPPEGs together with the acrylate concentration and molar mass calculated using 1H NMR. **Table S3.** Printing conditions (20 layers – for similar dimensions in swollen state taking in a count the swelling factor). **Table S4.** Info AUPPEG8K. **Table S5.** General patient details. **Table S6.** Patient characteristics of tumor samples. **Table S7.** Antibody list.

## Data Availability

"The mass spectrometry proteomics data have been deposited to the ProteomeXchange Consortium via the PRIDE partner repository with the dataset identifier PXD039590". The other datasets used and/or analyzed during the current study are available from the corresponding author on reasonable request.

## Abbreviations

^1^H NMR: Proton nuclear magnetic resonance
3D: Three-dimensional
AUPPEG: Acrylate-endcapped urethane-based poly(ethylene glycol
CAF: Cancer-associated fibroblasts
DMSO: Dimethylsulfoxide
ECM: Extracellulaire matrix
FTIR: Fourier transform infrared
GelMA: Methacrylated gelatin
HGSOC: High-grade serous ovarian cancer
HSP90: Heat Shock Protein 90
HSP90i: HSP90 inhibitor
kDa: Kilodalton
LGSOC: Low-grade serous ovarian cancer
MAPK: Mitogen-activated protein kinase
MEK: Mitogen-activated protein kinase kinase
MEKi: MEK inhibitor
MRI: Magnetic resonance imaging
PEG: Poly(ethylene glycol)
PLA: Poly-lactic acid
SEM: Scanning electron microscopy
TME: Tumor microenvironment
