# Supplementary material for: A preclinical platform for assessing long-term drug efficacy exploiting mechanically tunable scaffolds colonized by a three-dimensional tumor microenvironment
Source: Biomater Res. 2023 Oct 18;27:104. doi: 10.1186/s40824-023-00441-3 (PMC10583378; doi:10.1186/s40824-023-00441-3)
Supplement: Supplementary file 1 — Additional file 1: Fig. S1. Peritoneal metastasis scaffold model with SK-OV-3. Fig. S2. CAF in LGSOC tumors. Fig. S3. LGSOC 2D monocultures. [file 40824_2023_441_MOESM1_ESM.docx]

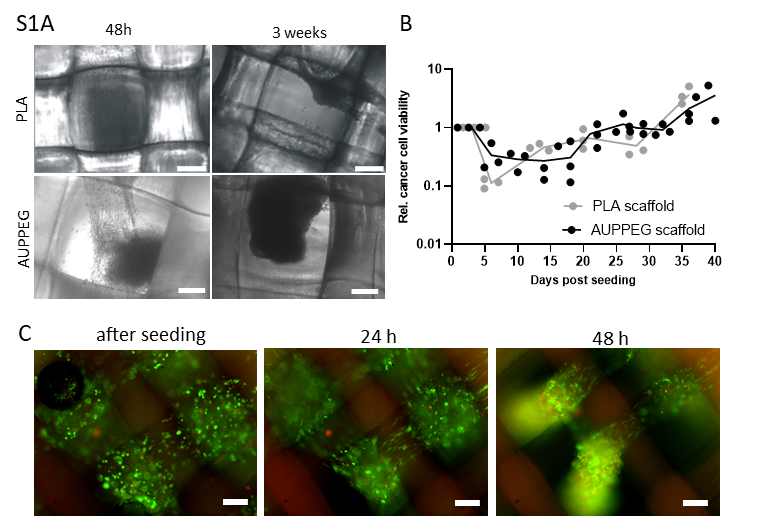


Fig. S1: Peritoneal metastasis scaffold model with SK-OV-3

A) Phase contrast images of SK-OV-3 Luc eGFP and CAF seeded on a PLA (historic data [11]) or a AUPPEG4K scaffold, 48h and 3 weeks after seeding. After 48h cells were organized in a spheroid and collagen fibers were visible between the struts of the AUPPEG scaffolds. Scale bar 100 µm. B) Relative cancer cell viability of SK-OV-3 Luc eGFP seeded on PLA (historic data [11]) or AUPPEG scaffold determined by BLI. C) Fluorescent images of SK-OV-3 Luc eGFP (green) and CAF (red) seeded on a AUPPEG4K scaffold, immediately, 24 h and 48 h after seeding. Round cells (after seeding) became elongated (24 h), which is an indication of migratory cells, and self-assemble into a spheroid (24 h). Scale bar 200 µm.


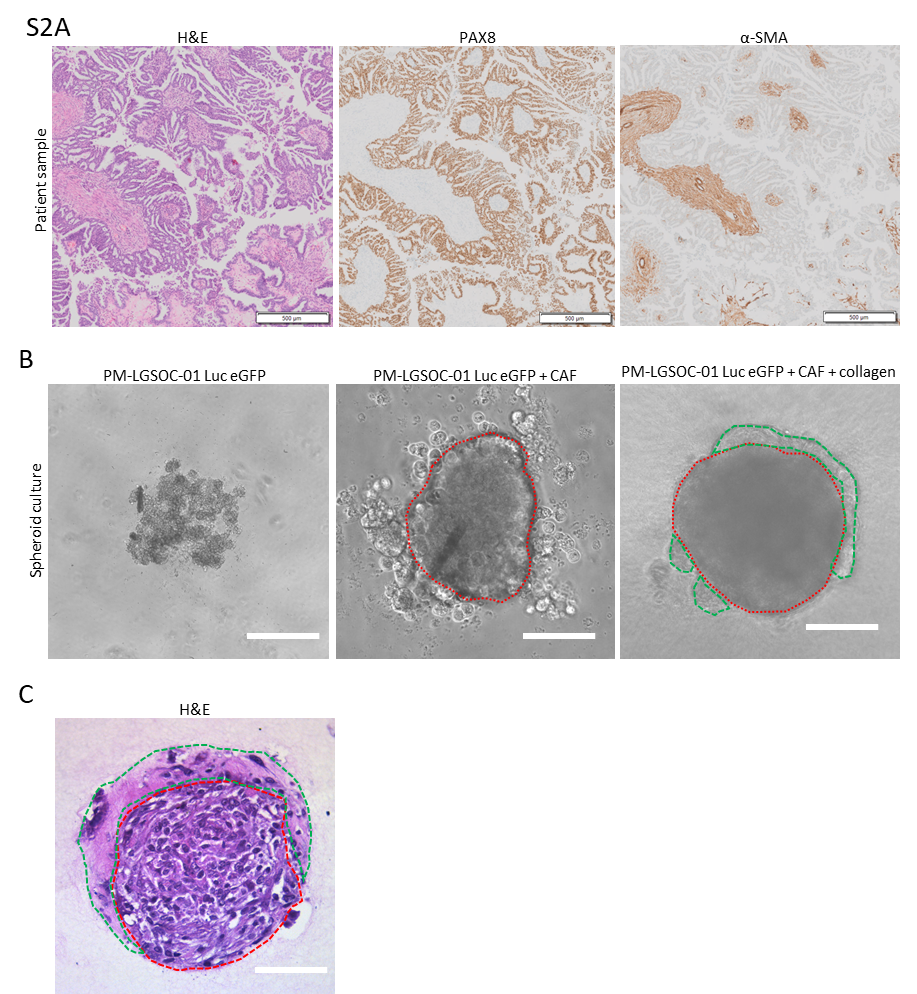


Fig. S2: CAF in LGSOC tumors

A) Hematoxylin & Eosin (H&E), PAX8 (ovarian cancer marker) and α-SMA (CAF marker) comparison of the original LGSOC patient sample. Scale bar 500 µm. B) Spheroid culture, LGSOC cells seeded on ultra-low adhesion plates as monocultures, co-cultures with CAF and co-cultures with CAF in the presence of type I collagen, 48 h past seeding. Red dotted line indicates CAF, green dotted line indicates LGSOC cells, scale bar 100 µm. C) H&E staining of LGSOC/CAF spheroid formed in the presence of type I collagen gel. Red dotted line indicates CAF, green dotted line indicates LGSOC cells, scale bar 100 µm.


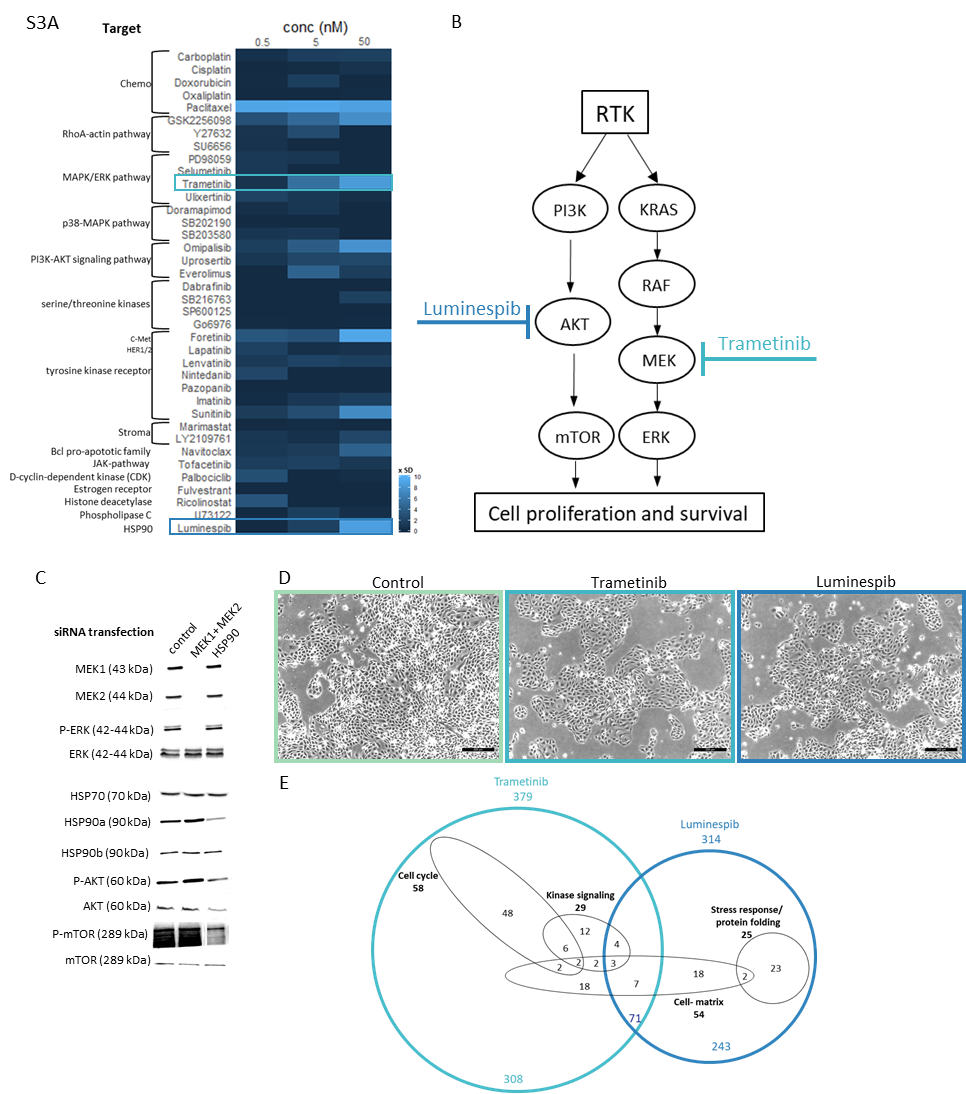


Fig. S3: LGSOC 2D monocultures

A) Heatmap of compound screening on 2D PM-LGSOC-01 cells. Color code indicates the impact of the compounds on cell growth evaluated by confluency monitoring by Incucyte® ZOOM. B) Pathway after activation of Receptor tyrosine kinases (RTK) leading to cell proliferation and survival. Sequentially phosphorylation of phosphoinositide 3-kinases (PI3K), RAC-alpha serine/threonine-protein kinase (AKT) and serine/threonine-protein kinase mTOR (mTOR) activates one branch of the pathway. The second branch is constitutively activated in the PM-LGSOC-01 cells by a mutation in KRAS gene leading to RAF proto-oncogene serine/threonine-protein kinase (RAF), dual specificity mitogen-activated protein kinase kinase (MEK) and Mitogen-activated protein kinase (ERK) activation. Both pathways complementary act to mediate proliferation and survival of cancer cells. C) Western-blot of cell lysates of PM-LGSOC-01 treated with siRNAs targeting MEK1, MEK2 and HSP90. Reduced protein expression impacts downstream signaling events as indicated by using phospho (P)-specific antibodies. D) Representative phase contrast images 72 h after siRNA treatment. Scale bar 200 µm. E) Proteins grouped by function affected by treatment with MEKi/HSP90i (Trametinib/Luminespib) evaluated by unbiased proteomic analysis 24 h post treatment of PM-LGSOC-01 cells.
