# Supplementary material for: A preclinical platform for assessing long-term drug efficacy exploiting mechanically tunable scaffolds colonized by a three-dimensional tumor microenvironment
Source: Biomater Res. 2023 Oct 18;27:104. doi: 10.1186/s40824-023-00441-3 (PMC10583378; doi:10.1186/s40824-023-00441-3)
Supplement: Supplementary file 2 — Additional file 2. Supplementary materials and methods [46–49]. [file 40824_2023_441_MOESM2_ESM.docx]

**Supplementary materials and methods**

Compound screening

Compound screening of 39 selected inhibitors / chemotherapeutics was performed on 2D cultures of PM-LGSOC-01 cells in 384 well plates, 2 x 10^3^ cells/well. 24 h post seeding cells were treated with 0.5, 5 of 50 nM inhibitors / chemo or serum control (0.1 or 10%). Screening was performed in triplicate. Cell growth was monitored by Incucyte® ZOOM (Sartorius), by imaging and analyzing confluency every 4 h for 8 days. The area under the curve (AUC) of the growth curves was used for analysis. Growth rates were considered significant lower if the AUC differs > 2x the standard deviation (SD) (of the 1% serum control) compared to the 10% serum control. Analysis and heatmap were constructed with RStudio.

Protein inhibition by siRNA

PM-LGSOC-01 cells were treated with siRNA’s with the Nucleofector® II Device and kit (Lonza, Bazel, Switzerland) with siRNA for MEK1 (HsMAP2K1_6 , 5100300699), MEK2 (HsMAPK2_6, 5102225097) and HSP90a (HSP90 CA-4, SI00075971) from Qiagen (Hilden, Duitsland). After 24 h cells were lysed with Laemmli lysis buffer for standard western blot analysis (table S7).

Proteomics

2D cultured PM-LGSOC-01 cells were treated for 24 h with either 0.1% DMSO (control), 1 nM Trametinib or 10 nM Luminespib. The cells were then detached, washed three times (PBS), pelleted and frozen (-80 °C). Each cell pellet contained approximately 10 x 10^6^ cells.

*Cell lysis and Protein digestion*: All chemicals were purchased from Sigma, unless stated otherwise. The cell pellets were resuspended in 1 mL lysis buffer (20 mM HEPES (pH 8.0), 8 M urea, 0.4% NP-40) and lysed using a point sonicator (40 Hz amplitude, 3 x 3 s ON/OFF, repeat cycle twice, Analis). The cell debris (e.g. DNA, membrane fractions) was precipitated by centrifugation (16,000 x g, 15 min at room temperature (RT)). The supernatants were transferred and the protein concentration was determined by Bradford assay (BioRad). The cysteine residues are reduced and alkylated using 5 mM dithiothreitol (DTT) (30 min at 55 °C) and 10 mM iodoacetamide (IAA, 15 min at RT in the dark). Amphipol A8-A35 (Apols, Anatrace) is added to a final concentration of 1 mg/mL and incubated for 10 min at room temperature. The samples are subsequently acidified with trifluoroacetic acid (TFA, Biosolve) to a final concentration of 1%. After a 10 min incubation at RT, the proteins were precipitated by centrifugation (16,000 x g for 10 min at RT) and the supernatants removed. The Apols-protein pellet was washed once with 0.1% TFA before suspending in 500 μL 50 mM triethylammonium bicarbonate (TEAB) (pH 8.0). Proteins were digested overnight with 2.0 μg trypsin (Promega, 1:100 ratio (w:w)) at 37 °C.

*Peptide clean-up and TMT-10plex labelling*: the peptide mixture was acidified the next day with TFA to a final concentration of 1% (pH < 3). After 10 min incubation at RT, Apols was precipitated by centrifugation (16,000 x g for 10 min at RT) and the supernatants were isolated. Based on the Bradford protein concentration, app. 50 μg peptide material was isolated from each sample and labelled with their corresponding TMT-10plex label (1 h at RT, 600 rpm). The reactions were quenched with NH_4_OH (0.25% final concentration) for 15 min at RT, 600 rpm. The ten fractions were combined and completely dried in the SpeedVac. The dried peptide material was then suspended in loading solvent A (2% acetonitrile (ACN, Organics), 0.1% TFA) and cleaned-up using MacroSpin columns (C18 material, Harvard Apparatus) with each centrifugation step at 2,000 *x g*, 2 min, RT. In short, the column was wetted with 400 μL ACN, equilibrated with 3 x 400 μL loading solvent A after which the samples were loaded, washed twice with loading solvent A and the peptide material is eluted using 60% ACN, 0.1% TFA. The eluent is then dried completely.

*Pre-fractionation*: the sample was resuspended in 300 μL loading solvent A and the peptide concentration was determined (app. 2 μg/μL) on a Lunatic instrument (Unchained Lab). 70 μg (35 μl) of peptide material was loaded onto an HPLC (Agilent 1100 series) equipped with a trapping column (5 μm C18 beads, 250 μm I.D. × 4 cm) and analytical column (3 μm, 250 μm I.D. × 15 cm), produced in-house (Reprosil-Pur Basic C18-HD, Dr Maisch). Separation was performed with a 100 min linear gradient from 100% solvent A’ (10 mM ammonium acetate (pH 9) in ddH2O) to 100% solvent B’ (70% ACN, 10 mM ammonium acetate (pH 9) in ddH2O). After 16 min, 1-min wide fraction collection started. A total of 12 vials was used, meaning that the loop restarted at position 1 after completing one cycle until seven rounds were completed (time point 98 min). Each fraction was vacuum dried and stored at -20 °C freezer.

*LC-MS/MS analysis*: The samples were suspended in 30 μL loading solvent A’’ (0.1% TFA in ddH2O /ACN, 98/2 (v/v)) only moments before analysis on Ultimate 3000 RSLC nano LC (Thermo Fisher Scientific) in-line connected to an Orbitrap Fusion Lumos mass spectrometer (Thermo Fisher Scientific). The peptides were first loaded on a trapping column (made in-house, 100 μm I.D. × 20 mm, 5 μm beads) using loading solvent A’’ and analyzed on a 200 cm long μPAC™ column (PharmaFluidics) with C18-endcapped functionality. Separation of the peptide material was done using a 90 min non-linear gradient going from 2-55% solvent B’’ (0.1% formic acid (FA, Biosolve) in ddH2O /ACN, 20/80 (v/v)), followed by an immediate increase to 99% solvent B’’. The column temperature was kept constant at 50 °C. The mass spectrometer was operated in data-dependent mode, automatically switching between MS and MS/MS acquisition for the four most abundant ion peaks per MS spectrum. Full-scan MS spectra (375-1500 m/z) were acquired at a resolution of 120,000 in the Orbitrap analyzer after accumulation to a target value of 2E5 and a maximum injection time of 100 ms. The number of MS/MS isolations was determined based on the accumulation time within a 3 s cycle time. Precursor ions with a minimum target value of 10,000 were isolated for fragmentation at a normalized collision energy of 30%. The C-trap was filled for maximum 500 ms to a target value of 100,000. The MS/MS spectra (200-2000 m/z) were acquired at a resolution of 45,000 in the Orbitrap analyzer.

*Database search*: Data analysis was performed with MaxQuant v1.6.3.4 [47] using the Andromeda search engine with default search settings including a false discovery rate set at 1% on the PSM, peptide and protein level. The mass tolerance for precursor and fragment ions was set to 20 and 4.5 ppm, respectively, during the main search. Enzyme specificity is set to trypsin, thus with cleavage C-terminal of arginine and lysine, also allowing cleavage at Arg/Lys-Pro bonds with a maximum of two missed cleavages. The MS/MS spectra were searched against those originating from the sequences of the human proteins in the Swiss-Prot/UniProtKB database from June 2019, being 20,231 protein sequences (www.uniprot.org). Variable modifications were set to oxidation of methionine and acetylation of protein N-termini. In all database searches, the matching between runs and second peptide search options was turned off. Quantification type was set to reporter ion MS2 and the preset TMT 10-plex settings were selected. The PIF option was allowed with a threshold of 75%.

*Data analysis*: after completion of the database search, the corresponding file was further processed in the Perseus software (v1.6.2.2) [48] using the normalized intensities of the individual TMT channels. First, the reversed hits and proteins only identified by site were removed as well as the potential contaminants. Second, we grouped the different channels by treatment and log2 transformed the intensities from the reporter ions to obtain a normal distribution. We then removed protein IDs with less than 100% intensities in at least one group and normalized the intensities by subtracting the median from each column. A two-way ANOVA with a 0.05 confidence interval was implemented to evaluate the effect of either Trametinib or Luminespib individually, or the effect of the combination of the two e.g. the interaction. Venn diagram was based on the functional enrichment analysis performed with g:Profiler [49].
