# Supplementary material for: A preclinical platform for assessing long-term drug efficacy exploiting mechanically tunable scaffolds colonized by a three-dimensional tumor microenvironment
Source: Biomater Res. 2023 Oct 18;27:104. doi: 10.1186/s40824-023-00441-3 (PMC10583378; doi:10.1186/s40824-023-00441-3)
Supplement: Supplementary file 3 — Additional file 3: Table S1. Overview of long term (> 4 weeks) in vitro 3D cancer models [50–67]. Table S2. Overview of the synthesized AUPPEGs together with the acrylate concentration and molar mass calculated using 1H NMR. Table S3. Printing conditions (20 layers – for similar dimensions in swollen state taking in a count the swelling factor). Table S4. Info AUPPEG8K. Table S5. General patient details. Table S6. Patient characteristics of tumor samples. Table S7. Antibody list. [file 40824_2023_441_MOESM3_ESM.docx]

**Supplementary tables**

**Table S1: Overview of long term (> 4 weeks) *in vitro* 3D cancer models**

| **Cancer type** | **Matrix** | **Stiffness (kPa)** | **Stromal cells** | **Time in culture* (days)** | **Therapy** | **Time follow-up (days)** | **Ref** |
| --- | --- | --- | --- | --- | --- | --- | --- |
| Breast cancer | PEG-fibrin hydrogel | 4 - 5 | NA | 28 | NA | NA | [50] |
| Breast cancer | PEG-fibrin hydrogel | 2 - 12 | Fibroblast | 28 | Doxorubicin  Paclitaxel | 2 | [51] |
| Breast cancer | Alginate–gelatin hydrogel | - | CAF | 30 | NA | NA | [52] |
| Breast cancer | - | - | HUVEC  Fibroblast | 30 | NA | NA | [53] |
| Breast cancer | Silk-fibrin scaffold | - | NA | 30 | NA | NA | [54] |
| Breast cancer | Alginate-collagen type I-Matrigel | - | NA | 30 | Doxorubicin | 5 | [55] |
| Breast cancer | Alginate | - | Primary tumor with stroma | 30 | Fulvestrant | 14 | [56] |
| Breast cancer | Gelatin-MA | - | - | 35 | Paclitaxel | 33 | [13] |
| Breast cancer | PEG–adhesion peptide hydrogel | 0.3 - 4 | NA | 40 | NA | NA | [57] |
| Chronic Lymphocytic Leukemia | Alginate – adhesion peptide hydrogel | 11 - 14 | NA | 28 | NA | NA | [58] |
| Chronic Lymphocytic Leukemia | P(HEMA-AEMA)-RGD hydrogel | - | MSC | 70 | NA | NA | [59] |
| Colorectal cancer | PEG-fibrin hydrogel | 0.4 – 3.5 | PDX-mouse stroma | 28 | NA | NA | [60] |
| Colorectal cancer | Chitosan-pectin | 1 - 8 | NA | 44 | NA | NA | [61] |
| Glioblastoma | Collagen type I | - | HUVEC | 70 | Temozolomide | 40 | [14] |
| Glioblastoma | Fibrin-gelatin | 22 - 27 | HUVEC/ pericytes | 56 | Temozolomide  P-selectin inhibitor (SELPi) | 7  28 | [15] |
| Head and neck cancer | HA fibers-fibrin-thrombin | - | Fibroblast | 49 | NA | NA | [62] |
| Hepatocellular carcinoma | Alginate–gelatin hydrogel | - | NA | 28 | Lenvatinib  Sorafenib  Regorafenib  Apatinib | 6 | [63] |
| Neuroblastoma | Matrigel | - | HUVEC | 35 | Retinoic acid | 21 | [64] |
| Non-small cell lung cancer | PEG-fibrin hydrogel | - | MSC | 35 | NA | NA | [65] |
| Ovarian cancer | PLA-collagen type I | 115,000 | CAF | 35 | NA | NA | [11] |
| Pancreatic cancer | PU-fibrionectin-collagein type I | 20 | stellate cells  endothelial cells | 28 | NA | NA | [66] |
| Prostate cancer | Silk-fibrin scaffold | - | NA | 50 | NA | NA | [54] |
| Prostate cancer | Alginate-Chitosan | - | PBL | 55 | NA | NA | [67] |
| Sarcoma | Decellularized bone | - | MSC | 28 | NA | NA | [68] |

*: after model is completed, CAF: cancer-associated fibroblast, HA: hyaluronic acid, HUVEC: human umbilical vein endothelial cells, MSC: mesenchymal stem cells, PBL: peripheral blood lymphocytes, PEG: poly(ethylene glycol), P(HEMA-AEMA): poly(2-hydroxyethyl methacrylate-co-2-aminoethyl methacrylate), PLA: polylactic acid, PU: polyurethane, RGD: arginylglycylaspartic acid

**Table S2:** Overview of the synthesized AUPPEGs together with the acrylate concentration and molar mass calculated using ^1^H NMR.

| **AUPPEGs** | **C_acr_ (mmol/g)** | **Molar mass (g/mol)** |
| --- | --- | --- |
| AUPPEG4K | 0.340 | 6,200 |
| AUPPEG8K | 0.166 | 12,400 |
| AUPPEG10K | 0.136 | 21,750 |

**Table S3: Printing conditions** (20 layers – for similar dimensions in swollen state taking in a count the swelling factor)

| **PEG backbone** | **4 kDa** | **8 kDa** | **10 kDa** |
| --- | --- | --- | --- |
| Nozzle size | G27 | G25 | G27 |
| Printing temperature (°C) | 60 | 85 | 100 |
| Printing speed (mm/s) | 250 | 600 | 300 |
| Strut distance (mm) | 0.55 | 0.5 | 0.45 |
| Strut thickness (mm) | 0.12 | 0.11 | 0.10 |
| Pressure (bar) | 5 | 5 | 5 |

**Table S4: Info AUPPEG8K**

|  | **Uncoated** | **GelMA coated** |
| --- | --- | --- |
| Gel fraction (%) | 69 ± 1 | 89± 2 |
| Mass swelling ratio | 7.0 ± 0.1 | 7.7± 0.6 |

**Table S5: general patient details**

| **Total number of patients** | 8 |
| --- | --- |
| **Age (mean, +/-SD)** | 57.9 +/- 7.3 |
| **Female; Male** | 3; 5 |
| **Previous chemotherapy** | 7/8 |
| **Previous radiotherapy** | 0 |
| **Previous intraperitoneal surgery** | 6/8 |

**Table S6: patient characteristics of tumor samples**

| Tissue type | No. of patients | No. of samples | Gender |  | Age  (avg, SD) | Previous chemo | Previous RT | Previous surgery° |
| --- | --- | --- | --- | --- | --- | --- | --- | --- |
| Mesothelioma | 1 | 9 | F |  | 53 | Yes | No | Yes |
| Mucinous colon cancer | 1 | 3 | M |  | 50 | Yes | No | Yes |
| Colon adenocarcinoma | 4 | 8 | F, M |  | 58  +/- 8 | Yes | No | Yes (2), No (2) |
| Epithelial ovarian cancer | 1 | 2 | F |  | 66 | No | No | Yes |
| PM of rectum cancer | 1 | 3 | M |  | 61 | Yes | No | Yes |

° only previous intraperitoneal surgery is taken into account

**Table S7: Antibody list**

| **Antibody** | **clone** | **Host** | **company** | **Catalog number** |
| --- | --- | --- | --- | --- |
| **Ki67** | 30-9 | Rabbit, monoclonal | Ventana | 790-4286 |
| **PAX8** | SP348 | Rabbit, monoclonal | abcam | ab227707 |
| **αSMA** | EP188 | Rabbit, monoclonal | abcam | AC-0H167RUO |
| **MEK1** | H-8 | Mouse, monoclonal | Santa Cruz Biotechnology | Sc-6250 |
| **MEK2** | A-1 | Mouse, monoclonal | Santa Cruz Biotechnology | Sc-6250 |
| **p-ERK** | E-4 | Mouse, monoclonal | Santa Cruz Biotechnology | Sc-7383 |
| **ERK** | K-23 | Rabbit, polyclonal | Santa Cruz Biotechnology | Sc-94 |
| **HSP70** | C92F3A-5 | Mouse, monoclonal | Enzo life Science | SPA-810 |
| **HSP90a** | / | Rabbit, polyclonal | Enzo life Science | ADI-SPS-771-J |
| **HSP90b** | / | Rabbit, polyclonal | ThermoFisher | PA3-012 |
| **p-AKT (ser473)** | 193H12 | Rabbit, monoclonal | Cell Signaling | 4058 |
| **AKT** | / | Rabbit, polyclonal | Cell Signaling | 9272 |
| **p-mTOR (SER2448)** | / | Rabbit, polyclonal | Cell Signaling | 2971 |
| **mTOR** | / | Rabbit, polyclonal | Cell Signaling | 2972 |
